# Supplementary figures and images for: From Gene Networks to Therapeutics: A Causal Inference and Deep Learning Approach for Drug Discovery
Source: Pharmaceuticals (Basel). 2025 Aug 30;18(9):1304. doi: 10.3390/ph18091304 (PMC12472719; doi:10.3390/ph18091304)

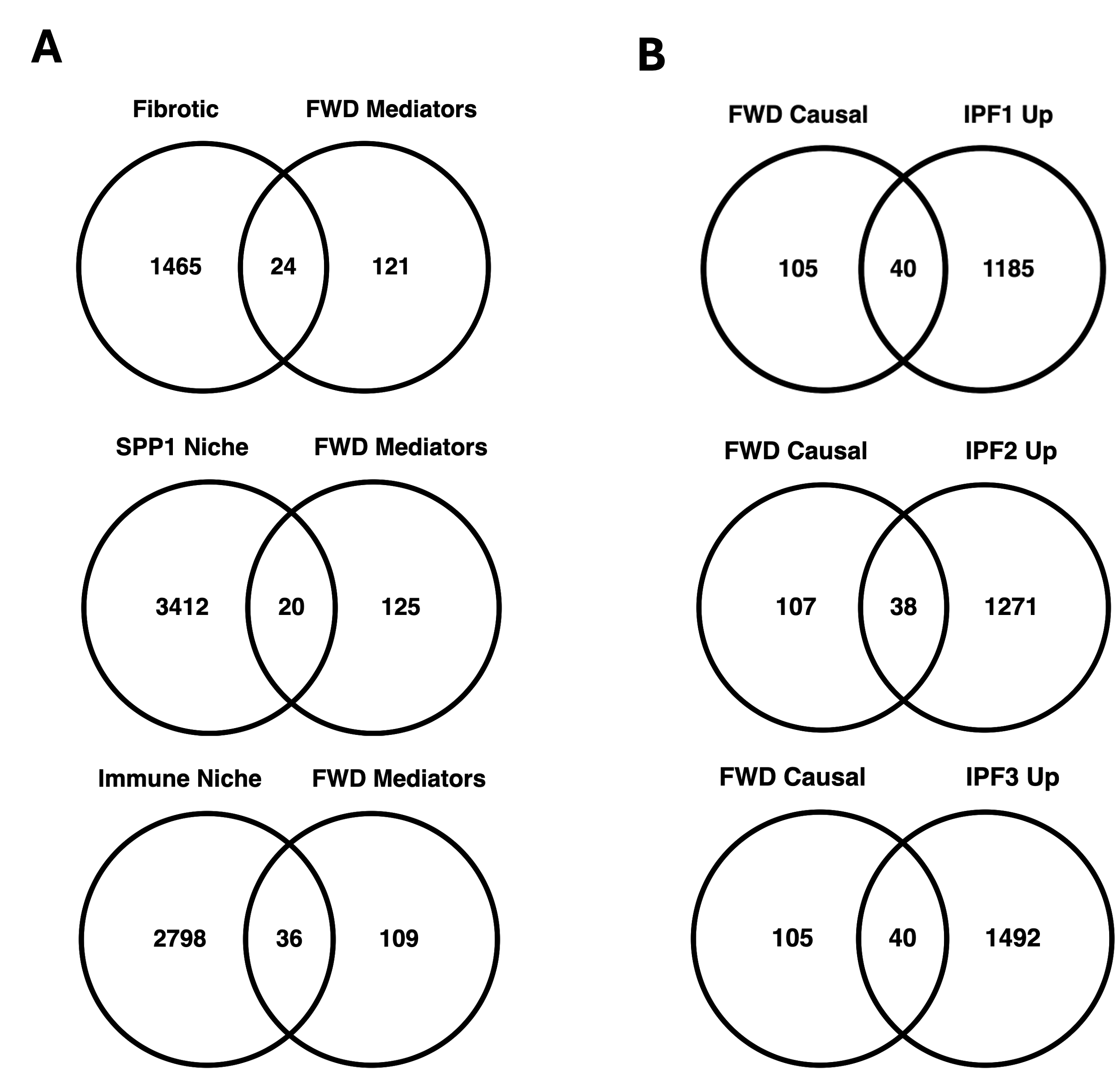

Supplement: Supplementary file 1 [file pharmaceuticals-18-01304-s001.zip › pharmaceuticals-3781314-Supplementary_Figure S1.png]

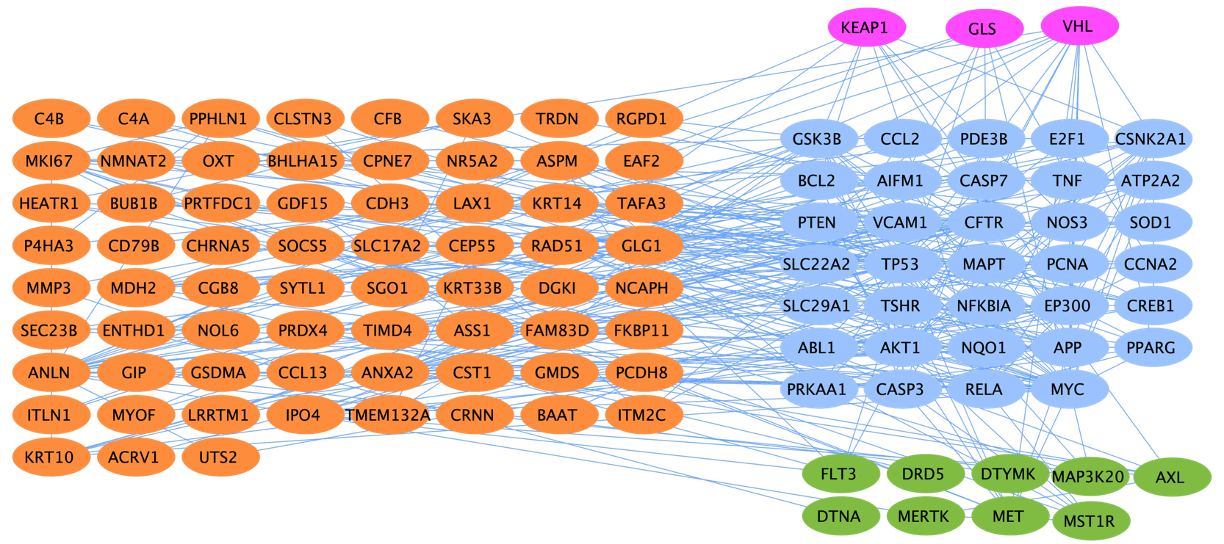

Supplement: Supplementary file 1 [file pharmaceuticals-18-01304-s001.zip › pharmaceuticals-3781314-Supplementary_Figure S2.png]
